# Application of component failure physics for the reliability assessment of an autonomous braking system

**DOI:** 10.1038/s41598-024-80476-1

**Published:** 2024-11-21

**Authors:** Debraj Banerjee, Cher Ming Tan, Nilim Akash Baruah

**Affiliations:** 1 Center for Reliability Science and Technology, Chang Gung University, No.259, Wenhua 1st Rd., Guishan Dist, Taoyuan City, 333323 Taiwan; 2 Department of Electronics Engineering, Chang Gung University, No.259, Wenhua 1st Rd., Guishan Dist, Taoyuan City, 333323 Taiwan; 3 Department of Radiation Oncology, Linkou Chang Gung Memorial Hospital, No.15, Wenhua 1st Rd., Guishan Dist, Taoyuan City, 333011 Taiwan

**Keywords:** Electrical and electronic engineering, Applied physics, Electronics, photonics and device physics

## Abstract

The growing demand for Cyber-Physical Systems (CPS) requires strong reliability. However, implementing Design for Reliability (DfR) in CPS requires a deep understanding of the components’ Physics of Failure. In this work, an autonomous braking system, a Cyber-Physical System is chosen to demonstrate the application of failure physics for DfR of CPS. The component under investigation is a crystal oscillator in the control circuit of the braking system. By subjecting it to a temperature cycling that mimics the realistic environment of the component, its degradation is found to significantly increase vehicle stopping distances, thereby posing potential safety hazards. Importantly, the relationship between the oscillator’s degradation and the stopping distance is non-linear, which is critical in avoiding simplistic extrapolations from initial degradation data to determine the time to replace the braking system. The outcomes of this study also provide essential design guidelines to enhance the reliability and safety of autonomous vehicle braking systems.

## Introduction

Advancements in integrated circuit technology, especially the more-than Moore, have enabled the miniaturization of sensors and actuators with excellent functionality, and it also enables reliable and high-speed communication protocols for information flow within an electronic system. Together with the development of powerful embedded systems to provide the computational capability for real-time processing and control within physical devices^1^, cyber-physical systems (CPS) evolved and became common. This CPS is a system that combines the virtual and physical worlds, seamlessly integrating computer-based algorithms and the physical components they control or monitor^2^.

Examples of cyber-physical systems are diverse and include smart grids, autonomous vehicles, industrial automation, smart buildings, and healthcare systems, and they are expanding to various domains that lead to the Internet of Things and Industry 4.0 that we are experiencing today^3^.

The increasing popularity of CPS stems from its numerous benefits as listed below^4^ :

1. **Increased Efficiency**: CPS allows for the automation and optimization of physical processes, leading to increased efficiency in various industries. For example, CPS can optimize production processes in manufacturing, reduce downtime, and improve resource utilization^5–7^.
2. **Real-time Monitoring and Control**: The ability of CPS to collect and process real-time data from physical processes enables continuous monitoring and control. This is critical in applications such as smart grids, where real-time adjustments can be made to ensure efficient energy distribution^5–7^.
3. **Improved Safety**: CPS enhances safety by providing real-time monitoring of physical systems and the ability to respond quickly to potential threats or malfunctions. This is particularly crucial in areas such as autonomous vehicles, where safety is paramount^5–7^.
4. **Autonomous Systems**: CPS enables the development of autonomous systems that can operate and make decisions without direct human intervention. This is seen in applications like autonomous vehicles, drones, and robots, where CPS plays a key role in navigation, perception, and decision-making^5–7^.
5. **Smart Infrastructure**: CPS allows for the efficient management of resources, such as energy and water, and enhances the overall sustainability of urban environments^5–7^.
6. **Healthcare Advancements**: In healthcare, CPS facilitates the integration of medical devices, patient monitoring systems, and information systems. This integration improves patient care by providing healthcare professionals with real-time data and decision support^5–7^.
7. **Enhanced Predictive Maintenance**: CPS offers significant advantages by facilitating real-time monitoring of equipment and machinery conditions. This capability aids in the early prediction and detection of potential issues, thereby preventing failures, minimizing downtime, and reducing maintenance costs^5–7^. In safety-critical systems, such as those utilized in nuclear power plants, CPS frameworks employ batch deterministic and stochastic Petri nets (BDSPNs)^8,9^ which are instrumental in evaluating dependability metrics, including performance and reliability. This approach supports the system reliability prediction during the design phase, which is crucial for implementing effective predictive maintenance strategies.

While CPS brings numerous benefits, it also poses challenges related to cybersecurity, privacy, and the ethical implications of autonomous decision-making^10^. Addressing these challenges is crucial for the responsible and sustainable deployment of CPS technologies. Another important challenge to CPS is its reliability. Any system without reliability will not be able to bring any tangible benefits it intended to, regardless of its functionality and capabilities, as it will not be dependable and soon will be replaced. There are several reported cases which bring disaster due to poor reliability of the system^11^.

In 2009–2011, Toyota recalled millions of vehicles due to unintended acceleration caused by faulty electronic throttle control systems^12–14^. The failure resulted in at least 89 deaths and 57 injuries^15^. In 1985–1987, a software bug in the Therac-25 medical linear accelerator caused six patients to receive massive overdoses of radiation, leading to three deaths and several injuries. The failure was attributed to poor software design and lack of safety interlocks^16^. In 2018–2019, two Boeing 737 MAX aircraft crashed due to a malfunctioning flight control system called MCAS, which repeatedly pushed the nose of the plane down based on erroneous sensor data. The crashes killed 346 people and led to the worldwide grounding of the 737 MAX fleet^17^.

Physical system reliability has been studied extensively in the past years, and theories of components to system reliability have been developed as can be seen in books written by Nelson^18^, Elsayed^19^, and Meeker^20^, to name a few. Given the importance of CPS reliability, reliability studies on CPS are also emerging extensively.

A CPS consists of software, hardware, and network connections. Hence, its reliability studies can be divided into the topics of networks, software, hardware, and human, as well as their interactions^21^.

In the area of network interaction and cascading failures in networks, Peng et al.^22^. explored the reliability of interconnected CPS by modelling the complexities of their network interactions and analysing the effect of cascading failures to improve system management. Peng et al.^23^. presented a new model for evaluating the robustness of interdependent CPS, using network and percolation theories to protect against and understand cascading failures, with strategies and simulations to enhance system resilience. They emphasize the importance of understanding complex system dynamics for enhancing resilience. This thread of enhancing system robustness through network theory is further explored by Zhou et al.^24^, who developed a reliability modelling method for cyber-physical distribution systems (CPDS), focusing on a multi-dimensional network model for the distribution information system (DIS) that incorporates the Fault Location, Isolation, and Service Restoration (FLISR) process. This DIS model considers communication network topology and information flow quality, assessing the reliability of DIS by evaluating multiple nodal attributes and their impact on the network’s state. They established a path search method and calculated network availability through Monte Carlo simulation. Along the same thread, Zhang et al.^25^, proposed an algorithm to calculate k-reliability for estimation of potential cascading failures of an entire system, highlighting the critical role of network structure and interaction in CPS reliability.

Specific to software reliability, Clarke et al.^26^ focussed on software reliability in transportation CPS rather than hardware and human factors. Their work suggested formal analysis techniques for software reliability. Faza et al.^27^ employed fault injection to simulate failure scenarios in Smart Grids, highlighting the role of software and algorithmic resilience in maintaining CPS functionality, particularly the Maxflow algorithm within the IEEE118 bus system. Ofori et al.^28^ examined the application of the Software Development Life Cycle (SDLC) for enhancing software reliability in CPS security, reviewing various security requirements engineering frameworks.

Focussing on CPS’ component reliability, Sanislav et al.^29^. introduced ReliaCPS, a multi-agent system that improves the reliability of cyber-physical systems by detecting and averting component failures, evidenced by a case study with positive impacts on vital reliability metrics. Nannapaneni et al.^30^ put forth a framework for assessing CPS reliability by framing it as a dependency issue, connecting software and hardware components to functional requirements. This framework is particularly adaptive at addressing sensor and software failures, facilitating runtime reconfigurations, as demonstrated through a smart parking system case study. This focus is shared by Castaño et al.^31^ who presented a case study on sensor reliability assessment in an autonomous driving scenario for the automotive sector where they designed a co-simulation framework by using an Internet of Things (IoT) LiDAR-based collaborative map to enable real-time interaction between virtual and real sensors, underscoring the significance of reliable components in overall system performance. Cai et al.^32^ also introduced a reliability analysis method for substations using a cyber-physical interface matrix, assessing the impact of failures in both physical and communication devices, particularly in the IEC 61,850 substation context.

Bessani et al.^33^ and Fan et al.^34^ extended the analysis to human factors and human-machine interactions, respectively, illustrating the multi-faceted nature of CPS reliability that encompasses not just technical, but also human elements. Bessani et al.^33^ presented a model to includes the operator’s responsiveness together with machines’ faults and failures to evaluate the reliability of a system. Fan et al.^34^ presented a platform and associated methodology to effectively generate accident scenarios by modeling human-machine interaction errors using model-level fault injection, followed by simulation to produce dynamic evolutions of accident scenarios.

The research of Yang et al.^35^ and Koc et al.^36^ further diversified the discussion, incorporating hybrid modelling approaches, communication failure assessments, and environmental adaptability of key components, reflecting the wide-ranging challenges faced in ensuring CPS reliability. Yang et al.^35^ presented an approach to assess CPS reliability and security, focusing on communication failures using an Instantaneous Availability model based on Markov processes, emphasizing the evaluation of communication system vulnerabilities. Koc et al.^36^ outlined a detailed reliability framework for CPS, assessing the reliability of key components such as computational units and communication networks, and testing the framework’s adaptability to different environmental conditions. Singh et al.^37^. proposed a reliability evaluation framework, categorizing failures into local, degrading, and catastrophic types, and suggest a protection system-like approach, modelling cyber interactions through probabilities of unreadiness or unwanted actions. They assessed the applicability of their method by reviewing established power system reliability techniques. Molnar et al.^38^. contributed to the reliability study of CPS by re-defining the boundaries of what constitutes a safety-critical system, proposing a method to categorize buildings for reliability assessment, and emphasizing the need for a comprehensive approach to reliability in Building Management System to ensure safety and functionality of various infrastructures.

In terms of reliability analysis methods, Zhou et al.^24^introduced a hybrid reliability modelling approach for CPS in distribution networks, combining fault trees and Petri nets to analyse system states under normal and cyberattack conditions, establishing new reliability indices. Wu.et.al^39^. introduced FARE (Failure Analysis and Reliability Estimation), a versatile framework for reliability evaluation of cyber-physical systems, offering a standardized approach across different CPS scenarios. It was validated on a New York City smart building system, proving its user-friendliness and precision, with prospects for setting industry benchmarks. Mitchell et al.^40^. presented a probabilistic model using Stochastic Petri nets to assess the reliability of cyber-physical systems (CPS) under various attack behaviours, with an intrusion detection and response system (IDRS) in place. They demonstrated that tuning the IDRS according to the detected attacker behaviour can significantly enhance CPS reliability. Various attack models were considered, and optimal settings for detection and response strengths were determined to balance energy conservation against intrusion tolerance, improving system reliability. The model helped to identify the most effective configurations for detection intervals, number of detectors, and response thresholds to maximize CPS reliability against persistent, random, and insidious attacks. Gong et al.^41^. focused on modeling the CPS as a Markov repairable system and using queuing theory to analyze its reliability. Wang et al.^42^. developed a simulation model to study the robustness of CPSs considering the interdependent relationship between nodes and links.

For reliability test and evaluation of CPS, Li et al.^43^ proposed a comprehensive reliability testing and evaluation strategy for CPS, considering both internal and external factors, and assessing component reliability across hardware, software, and architecture.

Leveraging the latest computational technologies, Yusupova et al.^44^. explored machine learning models to enhance CPS reliability, specifically employing long short-term memory (LSTM) neural networks for anomaly detection, designed to classify anomalies in CPS components. This technological frontier was further expanded by Rajawat et al.^45^, who applied deep learning to IoT node reliability in Smart Cities, aiming to enhance device and system performance through improved design, manufacturing, and maintenance strategies.

Significant progress has been made in the study of CPS reliability. The above-mentioned works evaluate the reliability of existing CPS, either as an entity by itself in the same way as system reliability methodology or considering its various parts within the CPS in the same way as component reliability. As it is well understood that if reliability can be incorporated during the design phase, significant cost reductions can be achieved. However, such Design for Reliability requires the knowledge of the Physics of Failure of the parts within a CPS system^46^. In this work, we approach the reliability study of CPS from the perspective of Physics of failure.

Castaño et al.^31^showed the various methods for the assessment of sensor reliability. While sensors are important in CPS, the degradation of other hardware components can also degrade the performance of CPS^47^, but this is rarely studied. The novelty of this work is to demonstrate a component in a control circuit besides a sensor that can also affect the performance or reliability of CPS. How the degradation of this hardware component will affect the software performance of CPS is also studied in detail, which is another novelty of this work.

In fact, the Reliability of CPS is unique in the sense that its reliability can be either due to the physical system alone, the embedded software alone, and/or the interaction of the physical system with computation algorithms^2^. A degraded physical system can render the functionality of the computation algorithm “degraded”, and vice versa. In this work, we create a hypothetical CPS to demonstrate how a degraded component (besides sensors) in a physical system can degrade the reliability of a CPS.

Specifically, we examine how a degraded oscillator can lead to significant timing errors, timing voltage over-shoot and under-shoot in the control circuitry of an autonomous braking system of vehicles. This delay, while seemingly insignificant on its own, translates into a substantial increase in stopping distances, potentially leading to hazardous situations. Through condition monitoring and the implementation of an alert management system, we aim to detect and analyse the impact of oscillator degradation on the autonomous braking system.

The paper is structured as follows: Section I provides the introduction to the reliability study of CPS. Section II describes a hypothetical CPS that is used to demonstrate the application of failure physics for the DfR of CPS. Section III details the experimental setup and procedures employed in this work; Section IV shows the interaction of the hardware degradation on the software algorithm that renders a degradation in the performance of CPS. Finally, Section V concludes the work by summarizing the key findings and their implications for the reliability of CPS along with suggestions for future research.

## Description of hypothetical case

As autonomous driving is of intensive research, and its safety is of crucial importance, we create a case study of an autonomous braking system and demonstrate how the braking distance increases as one of the electronic components in the system is degraded. Braking distance is a crucial safety parameter for a car. It is noteworthy that all the electronic components can degrade and thus the safety of a car could deteriorate differently with the degradation of different components.

Temperature cycling is a common environment for automotive electronics, and hence we intentionally subjected one component to temperature cycling to mimic its possible degradation^48–50^.

**Fig. 1:**
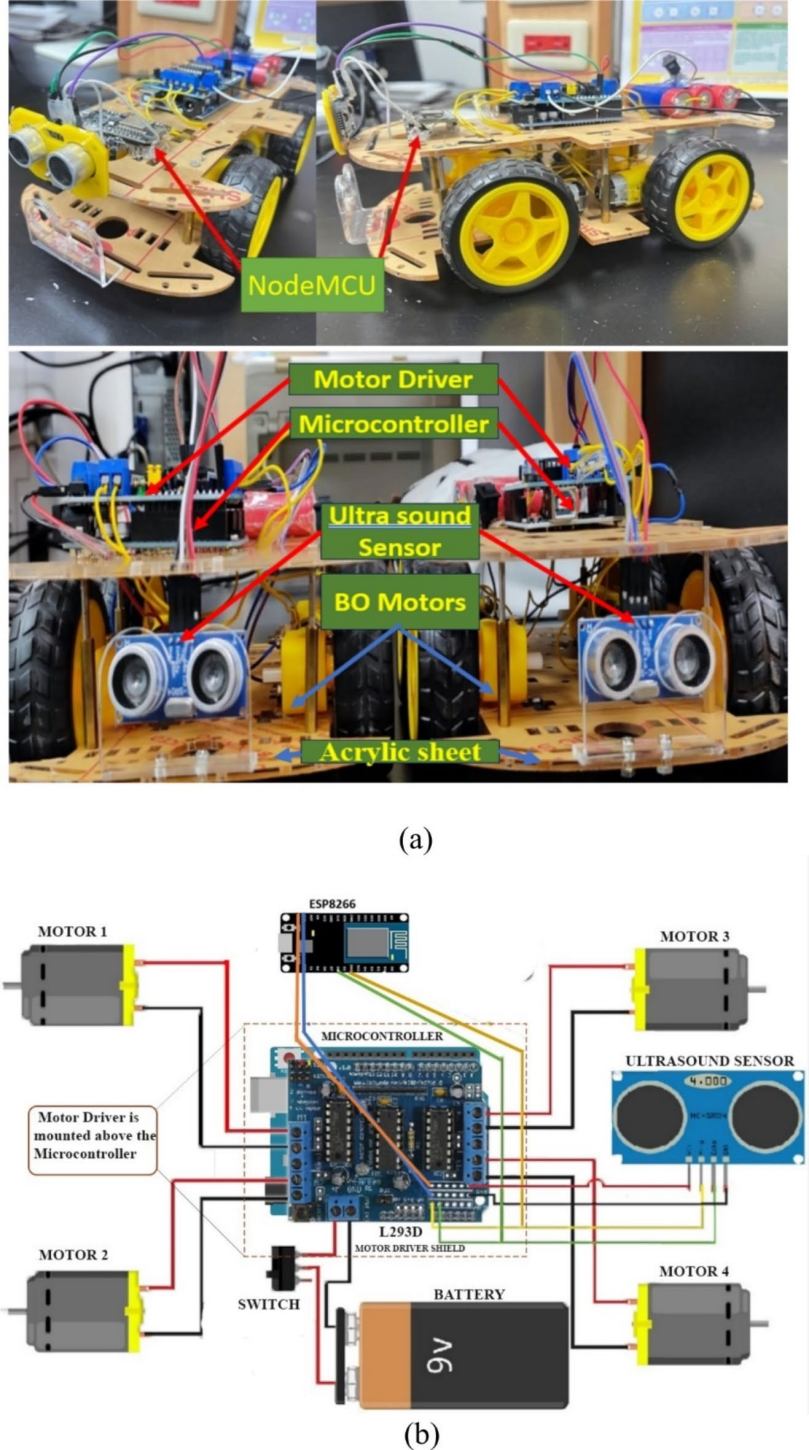
(a) Prototype for the autonomous cars; (b) Functional block diagram of the car prototype.

For demonstration purposes, we build a hypothetical autonomous car with autonomous braking system as shown in Fig. 1(a). The functional block diagram of the car is shown in Fig. 1(b). The car is constructed with a chassis made from an acrylic sheet measuring 19 cm in length and 12 cm in breadth, ensuring a sturdy yet lightweight foundation. 100RPM/3–12 V Battery operated DC motors are mounted onto this base and connected to an L293D motor driver. The core of the control system in each car is a microcontroller which is based on Arduino Uno open-source electronic prototyping platform. Arduino Uno is a microcontroller board that relies on the ATmega328 microcontroller chip. The ATmega328 is a low-power microcontroller with 8-bit data bus width and wide operating voltage of 1.8 to 5.5 V. It has 14 digital input/output pins; 6 of them can be used as PWM outputs, 6 analog inputs, ceramic, quartz crystal oscillator, USB connector, a power jack, an ICSP header, and a reset button^51^. The microcontroller is installed onto the L293D motor driver shield, which is also linked to an HC-SR04 ultrasonic sensor. To perform conditioning monitoring and to implement an effective alert system, NodeMCU ESP8266 Wi-Fi module 3.3 V/2.4 –2.5 GHz is integrated into the car’s design. The cars are powered by a set of three 2600mAh, 3.7 V DC NMC (nickel manganese cobalt oxide) lithium-ion (Li-ion) batteries connected in series.

Our hypothetical car is far from the actual autonomous car, and the components used are also different. As this work is to demonstrate how a degraded non-sensing hardware component can affect the reliability of a CPS, which is an autonomous braking system, the different hardware used in our system and the actual system will not affect the purpose of this work.

These cars (we build a total of 4 cars for experimentation) are programmed to move autonomously in the absence of obstacles. However, upon detecting an obstacle within a 60 cm range, the cars are commanded to halt. Its operation is described in the following.

The microcontroller interprets signals from the ultrasonic sensor to determine the presence and distance of obstacles. Based on this information, it instructs the motor driver to either continue the car’s movement or to stop it. The DC motors play a crucial role in propelling the car, responding adeptly to commands relayed through the motor driver from the microcontroller via the L293D shield which in turn processes data from the ultrasonic sensor^52^.

**Fig. 2:**
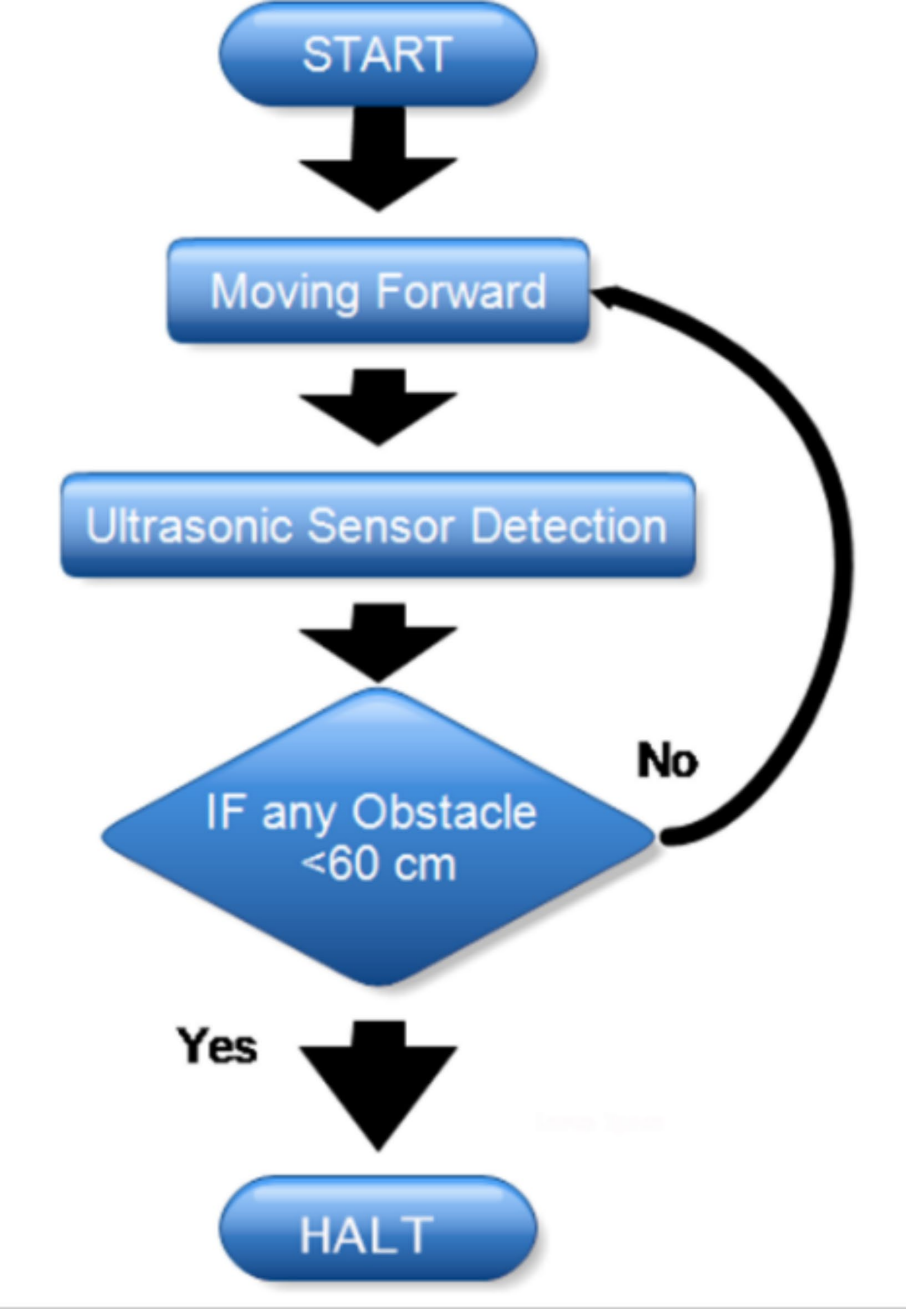
Flow chart for the commands given to the car.

The ultrasonic sensor plays a crucial role in detecting the presence and distance of obstacles in the path of our autonomous car^53^. This detection capability allows the microcontroller to process the distance data and make real-time navigational decisions about the car’s movements, such as when to stop to avoid collisions, as shown in the Flow Chart in Fig. 2.

As we can see from the flow chart, the braking distance is governed by a subsystem that consists of a microcontroller and motor driver. Microcontrollers are integrated circuits designed to govern a specific operation in an embedded system. These microcontrollers are central to the functioning of such embedded systems, combining processing power, memory, and programmable input/output peripherals in a single integrated circuit^54^. It also plays a vital role in processing and relaying instructions to the motor driver. L293D Motor driver is specifically designed for compatibility with the microcontroller boards, providing a seamless interface for motor control. It is integral to manage the car’s motors, responding to commands from the microcontroller, which in turn processes data from the ultrasonic sensor.

A critical aspect of a microcontroller’s operation is its timing mechanism, typically governed by a crystal oscillator^55^. For this microcontroller, a 16 MHz quartz crystal oscillator is used. This crystal oscillator gives a microcontroller a stable clock signal. This clock enables the microcontroller to communicate with other devices by sending and receiving signals accurately. The crystal oscillator determines the microcontroller’s clock frequency, thereby determining how fast it can execute a command.

As the oscillator is the heart of the operation of the microcontroller, and the microcontroller is the heart of the car, we choose the crystal oscillator as the component to be degraded. For automotive applications, it is common to have its components subjected to temperature cycling^48–50^, and hence we choose temperature cycling as the stress to degrade the oscillator.

## Experimentation

**Fig. 3:**
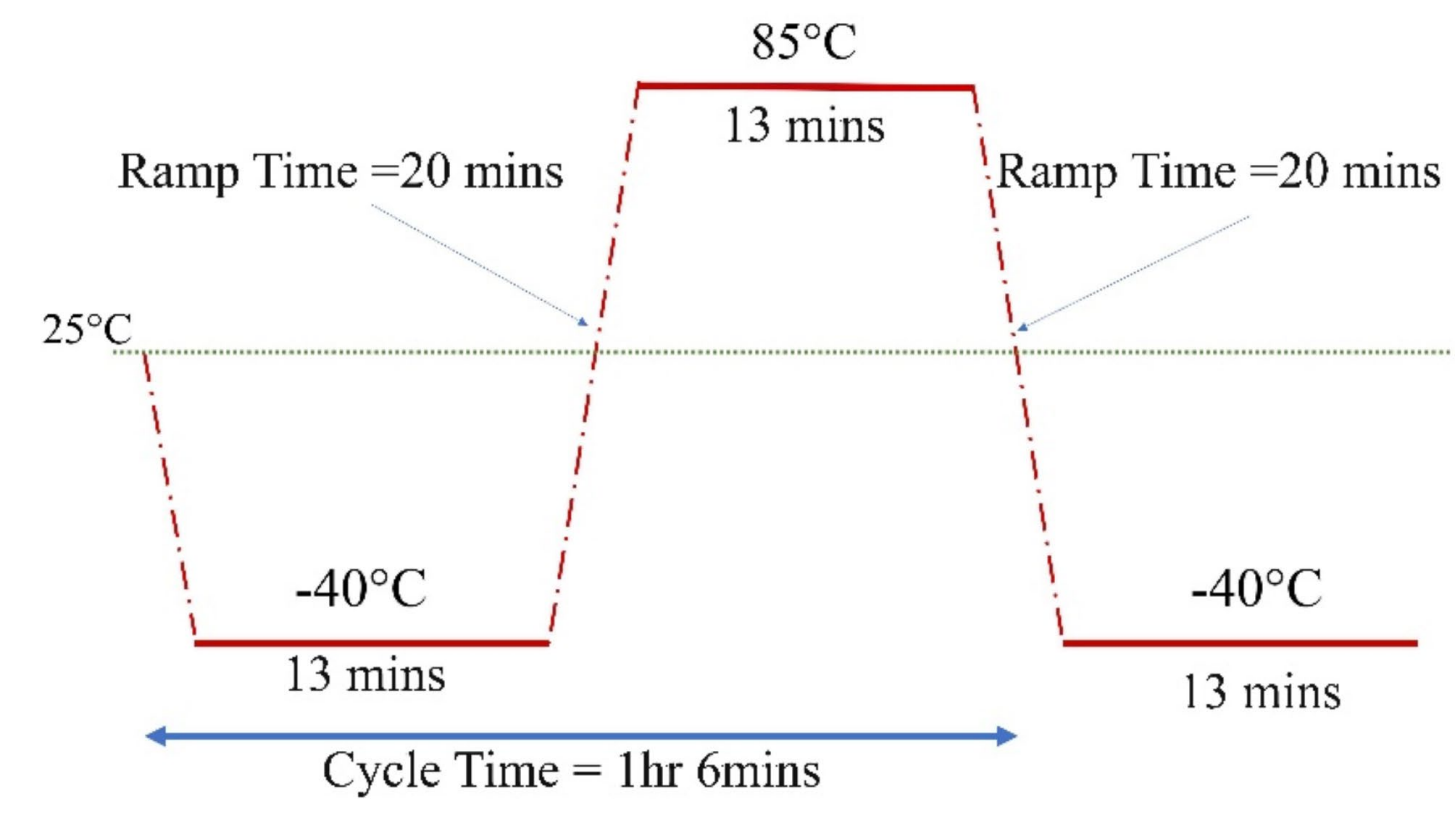
Temperature cycling condition for the testing.

As mentioned, 4 identical cars are made. One of them is used as a reference, and the other 3 will have their quartz crystal oscillators degraded by subjecting them to temperature cycling of various cycles up to a maximum of three weeks. The temperature cycling condition is as shown in Fig. 3, according to AECQ 200 level 3^56^. The total cycles that each oscillator is subjected to are tabulated in Table 1. We de-solder the oscillators first and put them in a temperature cycle chamber (model KD-162-FUL).

**Table 1:** Total number of cycles completed by each oscillator over sequential weeks.

Unit #	Total Cycle undergo
Oscillator #1	152 Cycles (Week1)
Oscillator #2	305 Cycles (Week2)
Oscillator #3	458 Cycles (Week3)

After the temperature cycling, the oscillators are re-soldered into the board to test the stopping distance of the cars. During this test, the microcontrollers are kept in a dry box to protect them from degradation due to the environment that could affect the experimental results. Great care is also taken when resoldering the oscillators back onto the microcontrollers, namely to keep the maximum solder temperature to 260^o^C and maximum solder time to 10 s^57,58^. The oscillator is allowed to have a maximum of two reflow soldering cycles according to manufacturing specification^59^, and hence the oscillator is de-soldered and then resoldered only once. This careful handling ensures that the change in the braking distance is indeed due solely to the degradation of the oscillators.

**Fig. 4:**
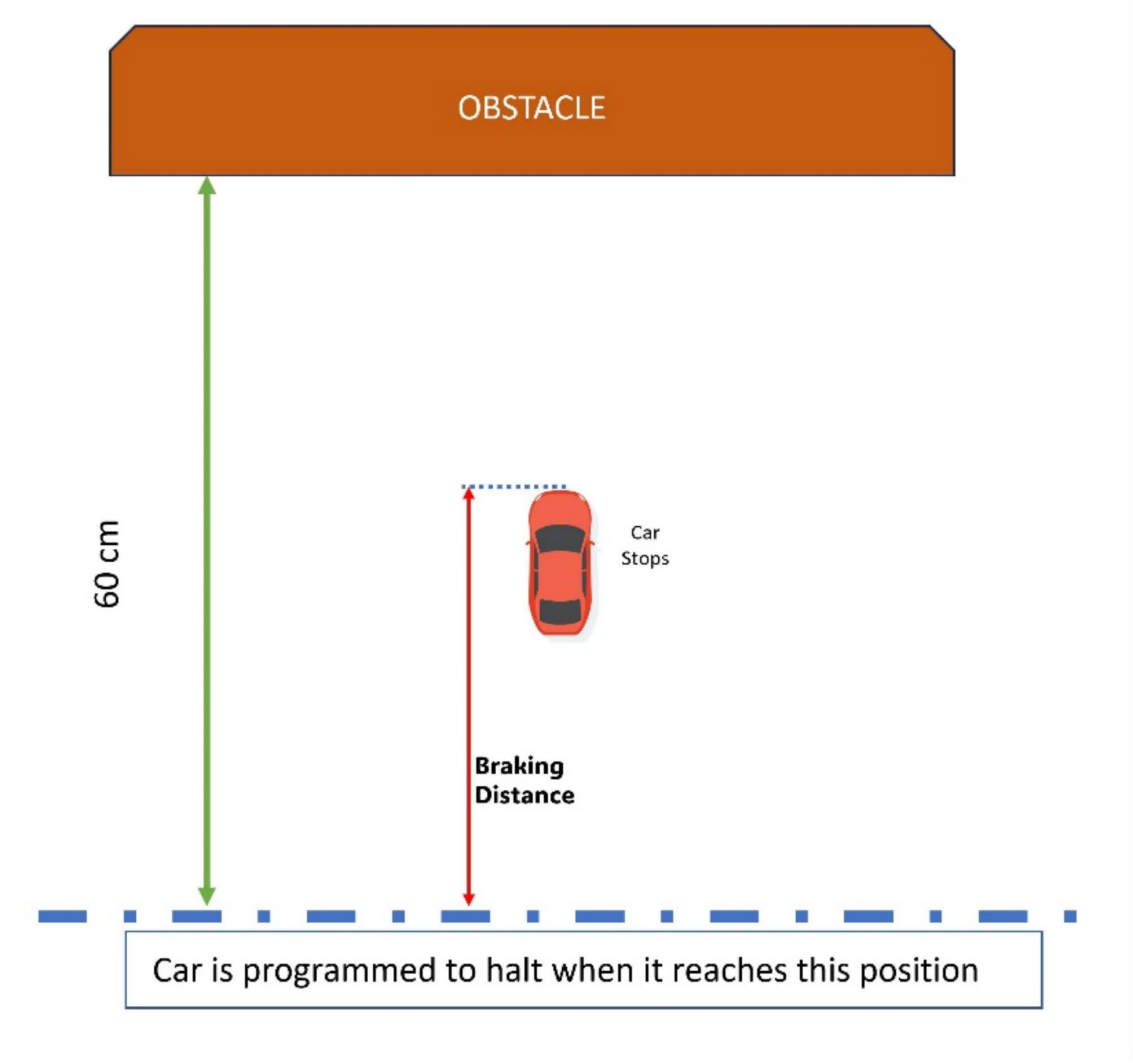
Explanation of the Braking distance.

Braking distance is defined in Fig. 4, it is the distance it travels after receiving the command to stop, measured from the point where an obstacle is detected at a distance of 60 cm (in this work, we program the microcontroller such that when there is an obstacle at a distance of 60 cm as measured from ultrasonic sensor, the microcontroller will issue a command to stop the car).

The braking distance experiment is performed at 2 different motor speeds by setting the duty cycle of the pulse width modulation (PWM) signal to 75% and 31% respectively. This PWM signal is fed to the motor for its moving and the average energy input to the motor can vary through the duty cycles of the PWM signal. A lower duty cycle implies lower power and hence a lower speed of the motor. The selection of two duty cycles in this work is to evaluate the performance of our autonomous braking system with different motor speeds.

A comprehensive approach to condition monitoring and alert management is developed for our CPS, particularly focusing on the implications of crystal oscillator degradation. After extensive testing with fresh and the 3 degraded oscillators, a critical threshold stopping braking distance was established using the car equipped with the fresh oscillator. For each car, the braking distance is recorded 20 times and the mean of the 20 records is considered as the braking distance of that particular car. This threshold serves as a benchmark for identifying potential degradation in the car’s braking efficiency. The alert management system enhances the car’s safety features by enabling real-time monitoring and immediate communication with the user, which is crucial for both reactive and preventive maintenance strategies.

**Fig. 5:**
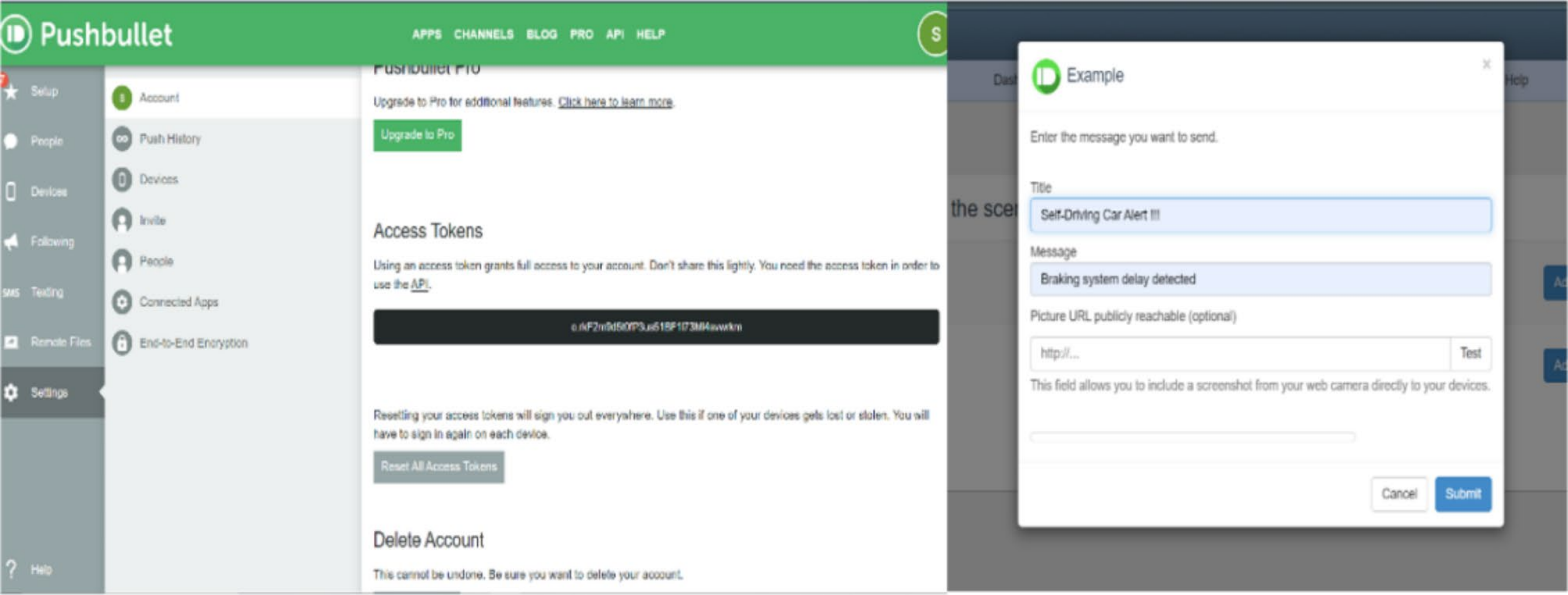
Pushbullet application for the alert management system.

For the alert management system, the data gathered by the ultrasonic sensor is transmitted using the NodeMCU to the user’s mobile device via an IoT application called Pushbullet^60^ as shown in Fig. 5. This application plays a pivotal role in monitoring the car’s braking system. It is specifically programmed to send alerts to the user if the car crosses the established threshold distance, indicating a potential degradation in the car’s braking performance. Pushbullet is a notification service chosen from a cloud-based platform known as Pushing Box, which will send various types of notifications to users, based on API calls. This versatile service allows a range of notification options such as Push notifications, SMS, Emails, or even Tweets, all enabled through a Wi-Fi connection.

The NodeMCU is then programmed to measure distance using an ultrasonic sensor and send alerts when a certain threshold is crossed. The NodeMCU includes necessary libraries for Wi-Fi and web server functionalities. The ultrasonic sensor’s trigger and echo pins are used for distance calculation. The Wi-Fi network credentials are specified for connecting the ESP8266 to the internet.

The code effectively integrates an ultrasonic sensor with Wi-Fi capability for remote monitoring and alerting. All the prototypes are programmed using the same code via the Arduino Integrated Development Environment (IDE). This programming directs the cars to operate based on instructions from a microcontroller.

The braking distances are meticulously recorded by the NodeMCU through the alert management system mentioned above. Based on the braking distance of the car with a fresh oscillator, a threshold limit of 16.46 cm is set. If the car exceeds the specified limit after an obstacle is detected, a notification is sent to the user via Pushbullet. Table 2 shows the difference in the braking distance of the cars with differently degraded oscillators.

**Fig. 6:**
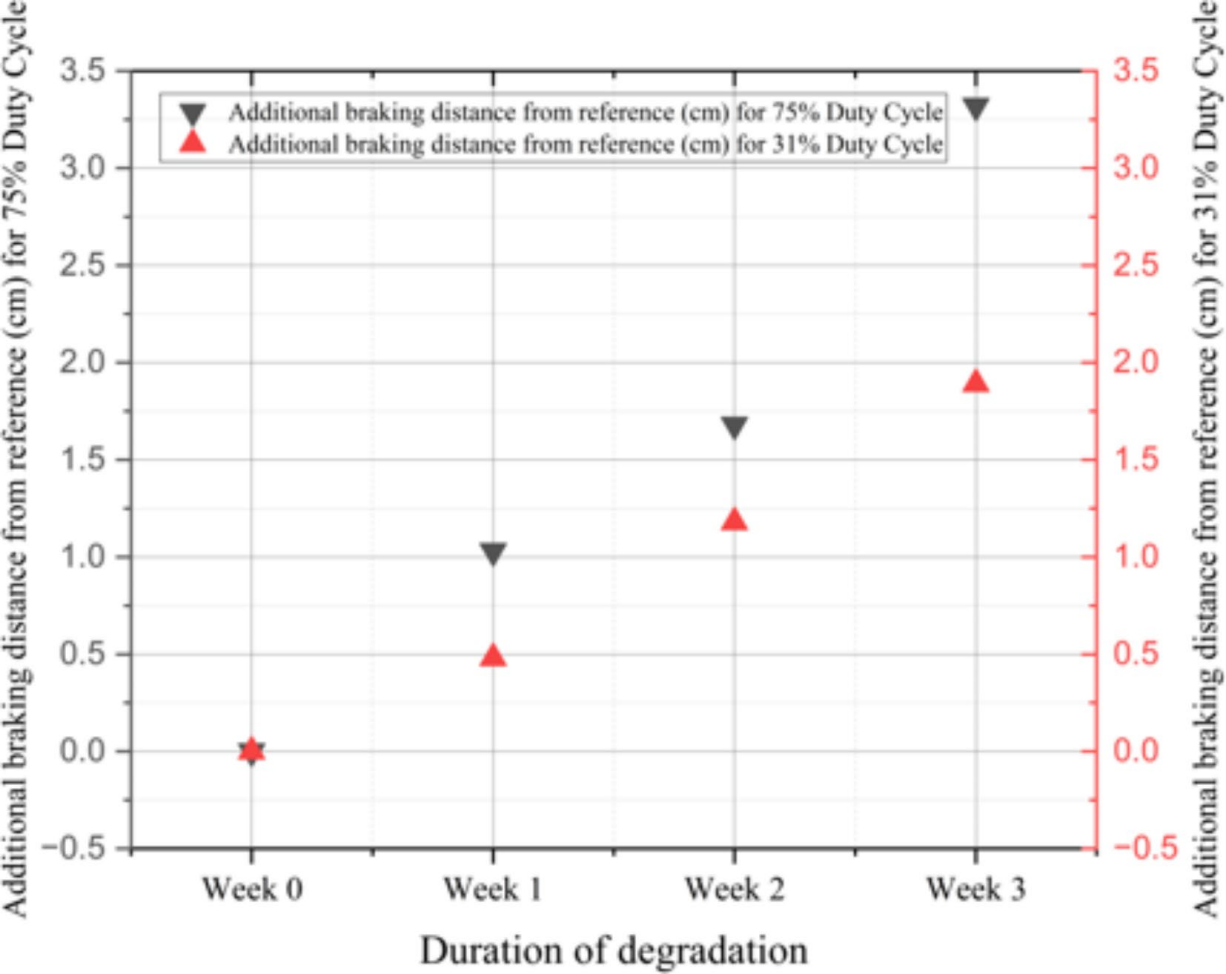
Additional braking distance from reference (cm) due to crystal oscillator degradation over three weeks duration at 75% duty Cycle and 31% duty cycle respectively.

The braking distances in Table 2 are plotted in Fig. 6 for clearer visualization. The trend shown in Table 2; Fig. 6 indeed highlights a significant and steady increase in braking distances at both duty cycle settings with the duration of the degradation of the quartz crystal oscillators as expected. The higher the speed, the larger the additional distance.

**Table 2:** Braking distance of the cars with differently degraded oscillators.

Car #	@75% Duty Cycle		@31% Duty cycle	
	Braking distance (cm)	Additional braking distance from reference (cm)	Braking distance (cm)	Additional braking distance from reference (cm)
Car #1 with a Non-Degraded Oscillator	16.46	0	7.08	0
Car #2 with degraded oscillator (Week 1)	17.49	1.03	7.56	0.48
Car #3 with degraded oscillator (Week 2)	18.14	1.68	8.26	1.18
Car #4 with degraded oscillator (Week 3)	19.78	3.32	8.97	1.89

It is also interesting to see that the increase in the braking distance has a larger increase from the oscillator degraded for 2 weeks to that degraded for 3 weeks, especially at higher speed. In other words, the increase in the braking distance is not linear with the degradation. This is an important information so that one cannot linearly extrapolate the additional braking distance from the early information. The mechanisms of the increase in the braking distance and its nonlinear increase with the degradation duration is investigated in detail as described in the following section.

## Mechanisms of the increase in the braking distance due to oscillator degradation

Understanding the mechanisms of the increase in the braking distance due to the degradation of the oscillator can help in the design for reliability to either mitigate or reduce the impact of oscillator degradation which is bound to happen in the prolong operation of an autonomous braking system. To begin, let us first examine the braking subsystem and the parametric change in the subsystem brought by the degraded oscillator.

In our case, for the car to stop or move, the command is given by the microcontroller to the motor driver L298N of the motor driver module^61^. The L298N motor driver facilitates the control of both the speed and direction of DC motors. The speed control is typically implemented through PWM. It works by rapidly switching the motor’s power supply on and off, thereby regulating the amount of energy supplied to the motor. The key to PWM is the adjusting of its duty cycle, which is the proportion of time the power is ‘on’ compared to the total cycle time. By varying the duty cycle, the amount of energy delivered to the motor is modulated, allowing precise control over its speed. On the other hand, for a given duty cycle, a lower PWM frequency will increase the actual on-duration for the motor, and the motor will hence be moving faster due to larger amount of energy being imparted onto it.

Besides the frequency and duty cycle to control the speed of the motor, the positive voltage of the PWM signal is another factor as higher amplitude will imply higher energy injected into the motor, and thus the motor will also move faster^62^. In this work, we measure the PWM signal from pin 3 of the microcontroller that control the motor driver. We program the microcontroller to generate a PWM signal on pin 3 and observe them on an oscilloscope.

When the ultrasound sensor receives an echo signal bounced back from an obstacle in front, the timing information on the trigger pulse and the received echo signal will be recorded by the ultrasound sensor^63^. This information will then be provided to micro-processor for computation of the distance from the obstacle to the car. If the distance is equal or below the pre-set distance (which is 60 cm in this work), the microcontroller will adjust the duty cycle of the PWM signal to slow down the motor speed that led to eventual stop.

**Fig. 7:**
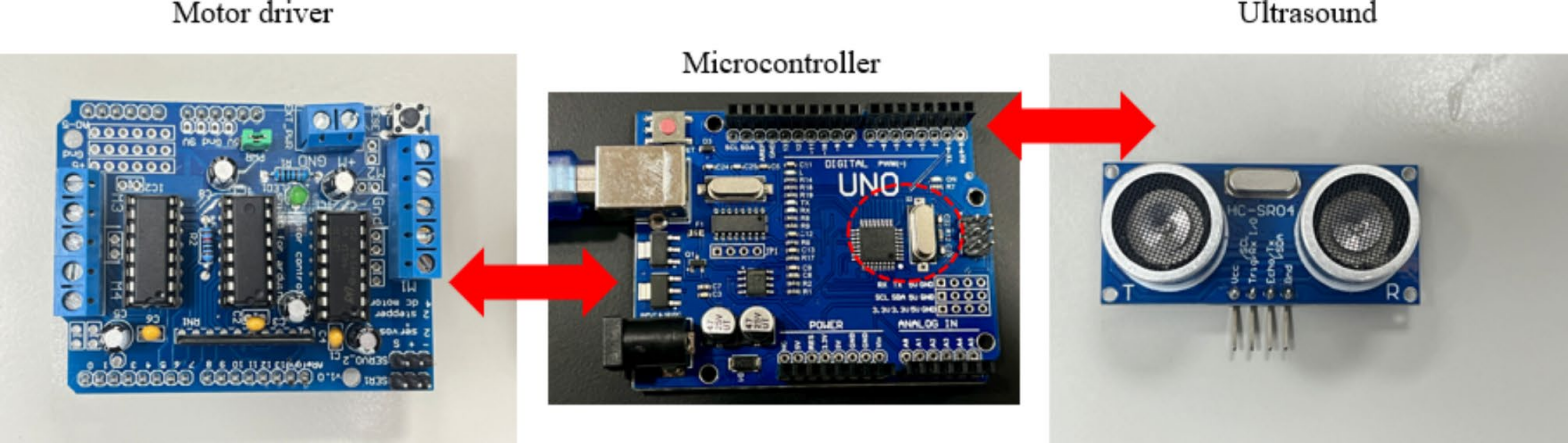
Schematic representation of the motor movement control mechanism.

The schematic of the above-mentioned stopping mechanism is shown in Fig. 7, which is also the mechanism of motor control mechanism.

Within the dotted circle, the left component is the microprocessor, and the right component is the oscillator.

With the above understanding of the mechanisms of motor moving and stopping actions, let us now examine the related parameters in the stopping mechanism due to the degradation of the crystal oscillator.

**Fig. 8:**
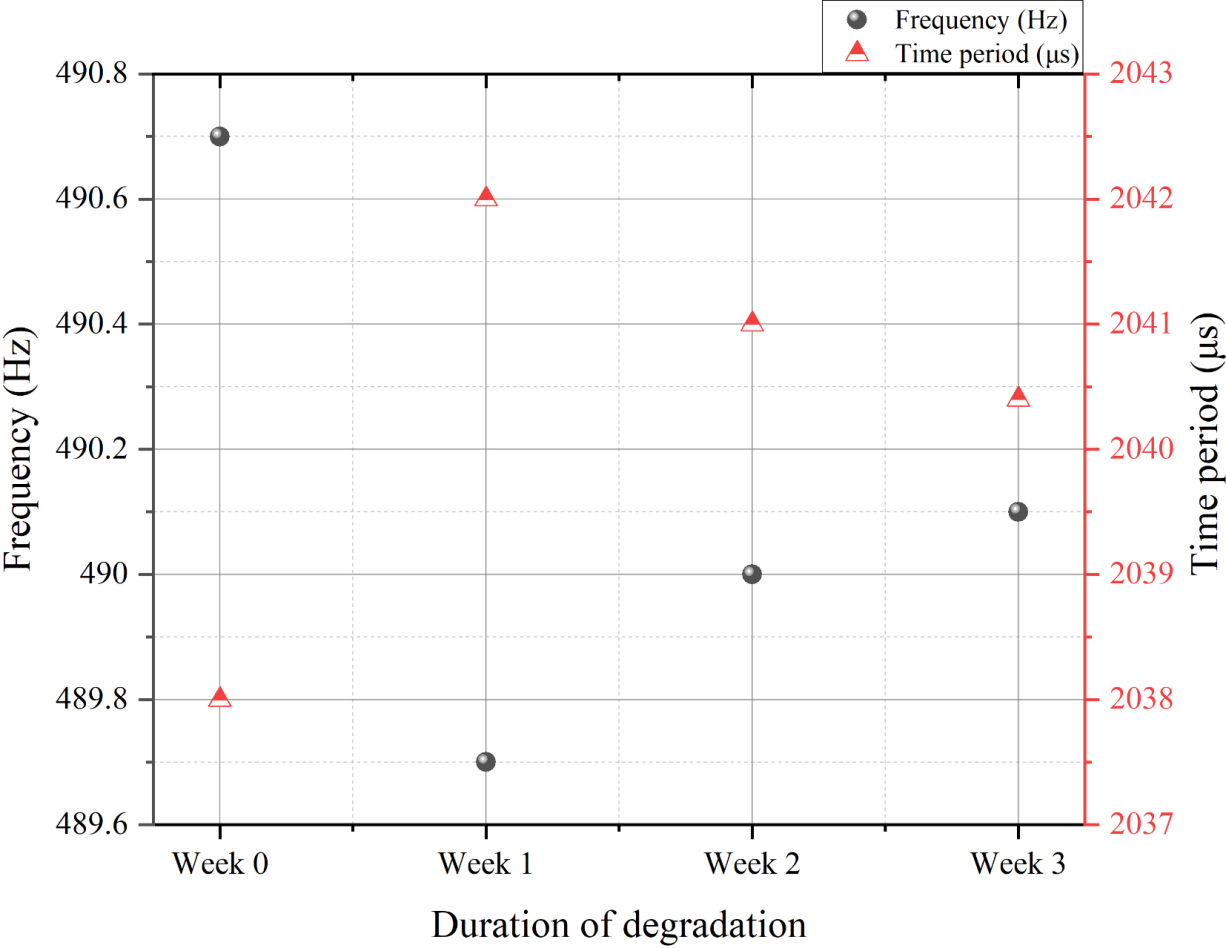
Shift in the PWM frequency from micro-processor to motor driver micro-controller pin 3.

Figure 8; Table 3 show the change in the PWM frequency and the time period of the oscillation from the micro-processor to the micro-controller for the motor driver IC circuit. The x-axis represents the week of degradation, and hence week 0 represents fresh unit. From Fig. 8, one can see an overall decrease in the PWM frequency with the degradation, but the decrease is the most for the 1-week degraded crystal. Since there is no intentional change in the duty cycle in our entire experiments for PWM, a decrease in the PWM frequency implies an increase in the period of supplying the energy to the motor, and thus the motor will move faster with the decrease of PWM frequency. For a given stopping power, this will render an increase in the braking distance as observed in Fig. 6. If the speed of the motor is set at a higher speed, i.e., at higher duty cycle, an increase in the period will imply a larger increase in the energy impart to the motor, and thus the braking distance will be increased even more as observed in Fig. 6.

**Table 3:** PWM frequency and period from degraded crystal oscillators.

	Fresh crystal 0 week	1-week degraded Crystal	2-week degraded Crystal	3-week degraded Crystal
Frequency (Hz)	490.7	489.7	490	490.1
Period (μs)	2038	2042	2041	2040.4

As can be seen in Fig. 8, the reduction in PWM frequency becomes smaller after the 1-week degraded crystal, and if this is the only mechanism for the increase in the braking distance, then the increase in the braking distance should be decreasing with the degradation duration of the crystal as the decrease in frequency becomes smaller after the 1-week degradation as observed in Fig. 8, but this is not observed in Fig. 6. Another factor, the shape of the PWM signals need to be considered.

**Fig. 9:**
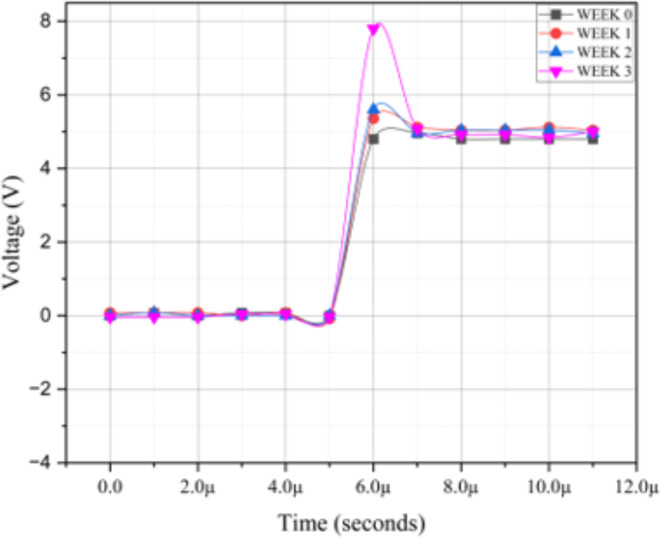
Overshoot in PWM signal due to degraded crystals.

Figure 9 shows the positive overshoot of the PWM signal. The overshoot increases for all the degraded crystals, but the overshoot becomes very large for the 3-week degraded crystal. As this overshoot also causes the motor to move faster, this renders an increase in the braking distance. The increase of the overshoot of 1-week degraded crystal from the fresh one is higher than that of 2-week degraded crystal from the 1-week degraded crystal, and together with the lesser decrease in the PWM frequency for 2-week degraded crystal, the incremental increase of the braking distance from fresh to 1-week degraded is larger than that from 1-week degraded to 2-week degraded crystal as observed in Fig. 6; Table 2 (1.03 vs. 0.65). However, the overshoot of 2-week degraded crystal is larger than 1-week degraded in comparison to the fresh one, the braking distance for 2-week degraded crystal is still larger than the fresh one and the 1-week degraded crystal. In other words, the impact of overshoot on the braking distance is much higher than the change in the PWM frequency, as the change in frequency is smaller for the 2-week degraded crystal in our work.

Along the same line of argument, as the overshoot in the 3-week degraded crystal has a drastic increase from 2-week degraded crystal as seen in Fig. 9, the significant increase in the braking distance of 3-week degraded crystal as observed in Fig. 6 can be understood.

**Fig. 10:**
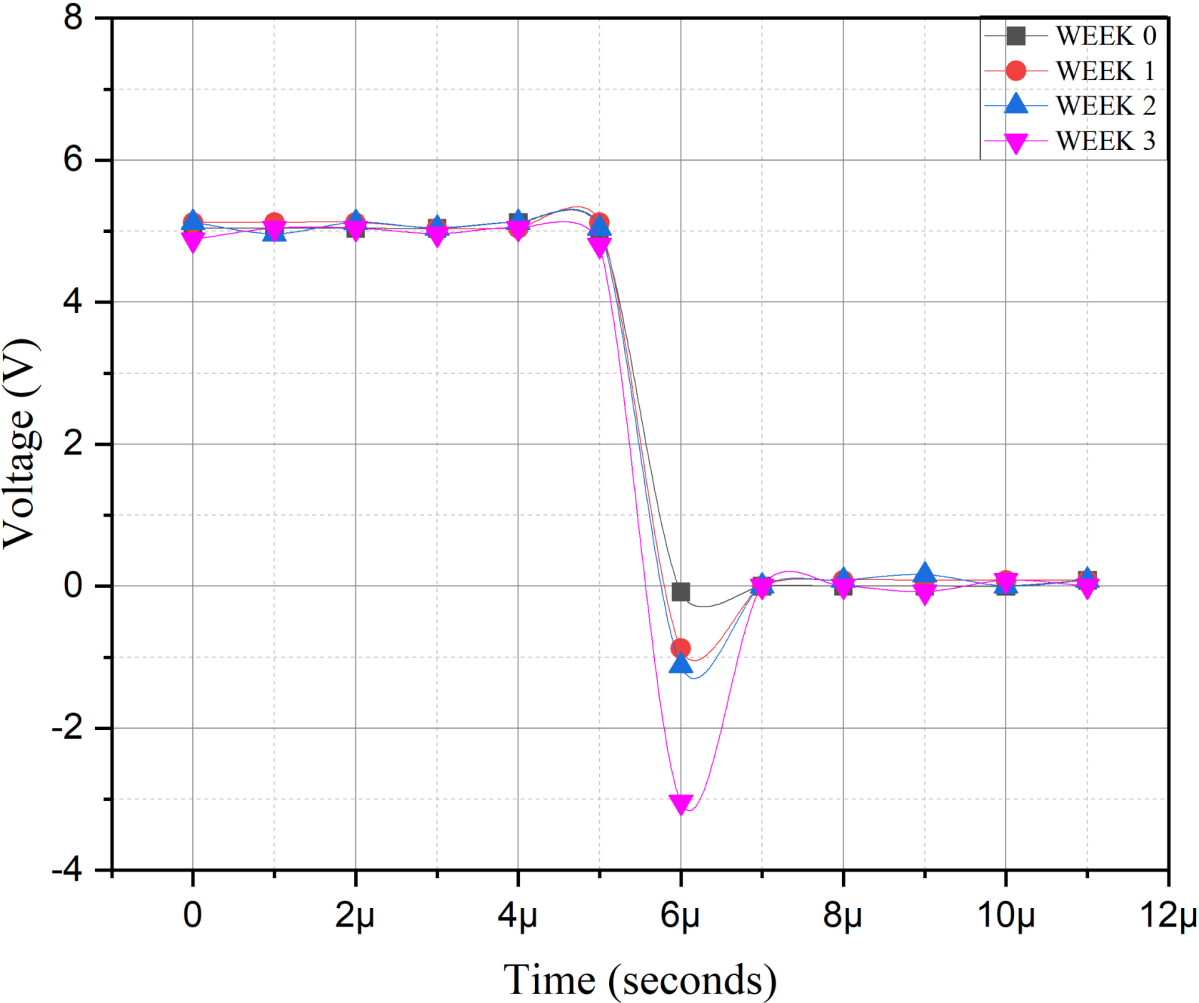
Undershoot in PWM signal due to degraded crystals.

As we look at the overshoot, we also examine the undershoot, and Fig. 10 shows the similar situation as overshoot.

Another characteristic of the shape of the PWM signal is the undershoot. To understand the impact of undershoot, we examine the motor driver IC L293D. It is a dual-channel H-Bridge motor driver that can control two DC motors or a single stepper motor^64,65^. A H-Bridge is a type of circuit that allows a voltage to be applied across a load in either direction. These circuits are commonly used in motor control, allowing for both speed and directional control. For DC motors as in our work, the speed is directly proportional to the applied voltage. Therefore, if the voltage increases, the speed of the motor will also increase. Hence, when there is an overshoot in voltage, the motor driver may apply a higher voltage to the motor for a brief period. This can cause the motor to spin faster than intended, as discussed earlier.

The presence of undershoot presents as a transient voltage spike can create what is called “shoot-through” in H-Bridge circuits, a condition where high current flows directly from the supply to ground through both the high-side and low-side switches that are momentarily on simultaneously^66^.

A simulation is performed to check the current increased or decreased for the overshoot and undershoot by using the measured overshoot and undershoot voltages that we obtained from fresh, 1-week degraded, 2-week degraded, and 3-week degraded crystals as input to the H-Bridge circuit as shown in the Table 4. The simulation is performed on an inverter circuit (the input circuit of H-bridge circuit), and a 3pF load capacitance is added on the output of the inverter circuit.

**Table 4:** Overshoot and undershoot characteristics of the PWM signal.

	Time Period	Frequency (Hz)	Overshoot		Undershoot	
	Micro Second		Actual Overshoot (volt)	V_maximum (volt)	Actual Overshoot (volt)	V_minimum (volt)
Week 0	2038.00	490.70	0.32	4.80	0.32	−0.08
Week 1	2042.00	489.70	0.48	5.36	0.96	−0.88
Week 2	2041.00	490.00	2.88	5.60	1.36	−1.12
Week 3	2040.40	490.10	2.96	7.80	3.20	−3.04

**Fig. 11:**
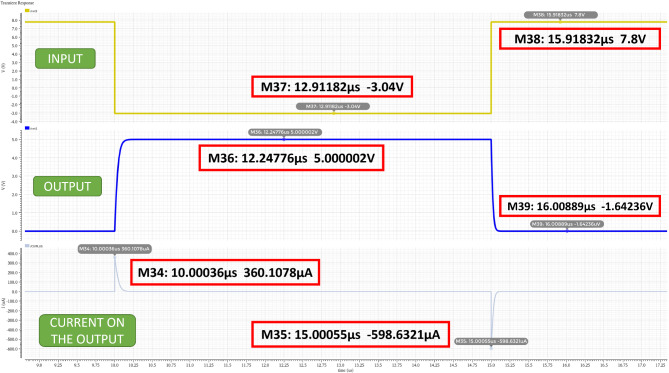
Simulation results of shoot-through for 3-week degraded crystal.

Figure 11 shows our simulation results for the case of using 3-week degraded crystal (for other degraded crystal, the results are similar except for lower current), and Table 5 provides the summary of the high current obtained from the simulation.

**Table 5:** Shoot-through current at the input of H-Bridge circuit due to PWM overshoot and undershoot voltages.

Weeks of degradation	Input Voltage (V)		Current through the MOSFETs (µA)	
	HIGH	LOW	HIGH	LOW
Week 0	4.80	−0.08	322.62	−385.46
Week 1	5.36	−0.88	343.03	−449.01
Week 2	5.60	−1.12	347.29	−479.77
Week 3	7.80	−3.04	360.11	−598.63

The presence of the shoot-through can degrade the motor driver, render further reliability issue of another electronic component, the motor driver IC. It also signifies that the MOSFET does not turn off immediately when the PWM signal goes to low level (presumable zero). In other words, the motor will be still spinning, and this will make the average speed of the motor higher since the duration of spinning is longer. Consequently, the increase in the braking distance increases drastically for 3-week degraded crystal as the corresponding PWM overshoot and undershoot becomes much higher. If this persists, the braking distance is expected to grow in an exponential way, presenting a hazardous situation. One should therefore not assume the degradation or the increase in the braking distance will proceed in a linear manner. The on-set of drastic increase should be well established to prevent hazardous situation from occurring.

From the understanding of the underlying physical mechanisms on the increase in the braking distance due to just a crystal degradation, the design of the braking system should therefore be the following to ensure the reliability and safety of the system:

- Monitor the change in PWM signal frequency and provide feedback to ensure its stability.
- Monitor the overshoot and undershoot voltage of PWM signal and provide feedback to limit the overshoot and undershoot voltages. This is more important as compared to (a).

**Fig. 12:**
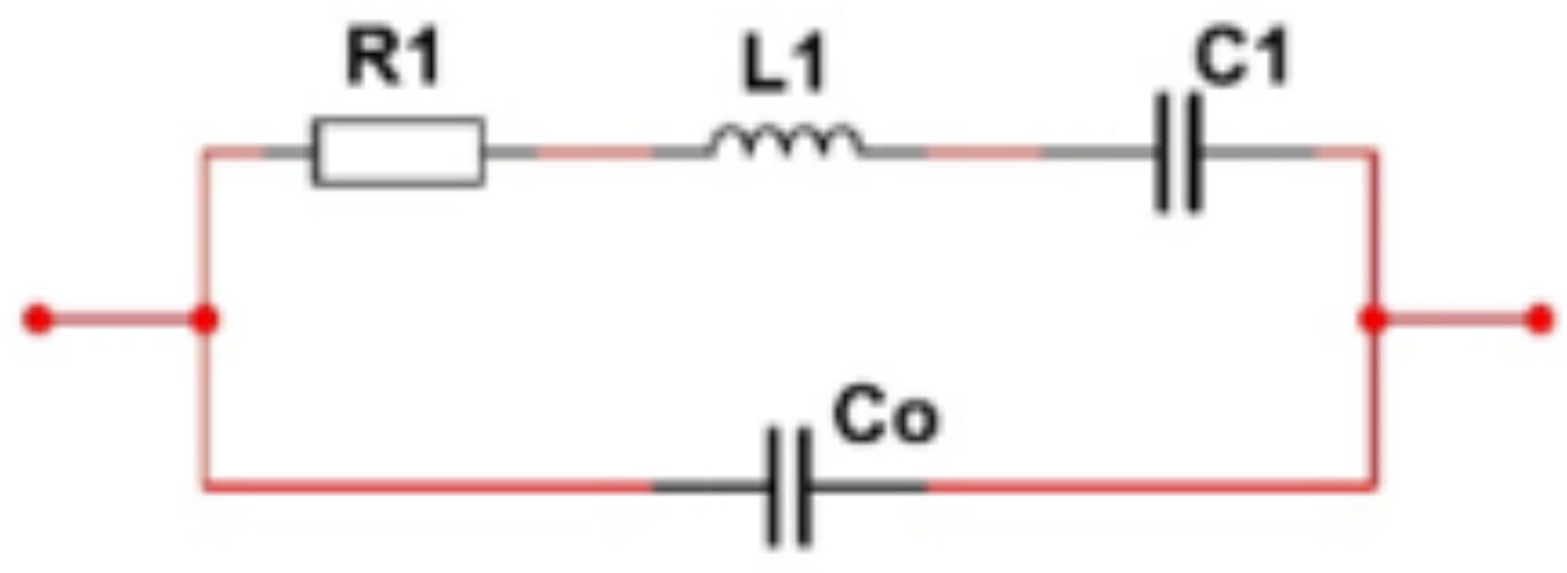
Equivalent electrical circuit of the crystal oscillator.

How exactly the degradation of crystal can lead to overshoot and undershoot depend on the PWM generation circuit, which is confidential in general. Fundamentally, such a phenomenon is related to the impedance mismatch of the crystal electrical parameters and the circuit electrical parameters. The electrical equivalent circuit of the crystal studied in this work is shown in Figs. 12^67,68,69^. The degradation of the equivalent electrical parameters of the crystal can be seen in Table 6. The time evolution of the degradation of these parameters is important for us to relate the 3-week degradation to the actual operation time of the crystal, for a given operating condition, as such evolution allows us to determine the acceleration factor of our temperature cycle test. Such acceleration factor can be derived either through monitoring the degradation of the equivalent electrical parameters and thus an empirical model can be obtained, or it can also be derived from the physical mechanisms of their degradations through detail failure analysis of the crystal degradation, and this will be reported in our next work.

**Table 6:** Degradation of the equivalent electrical parameters of the crystal.

	Inductance(H)	Capacitance(nF)	Resistance(Ω)
Non-Degraded (Week 0)	0.10	2.30	4.10
Degraded (Week 1)	0.09	2.81	4.34
Degraded (Week 2)	0.08	3.16	4.78
Degraded (Week 3)	0.07	3.55	5.23

## Conclusion

The impact of the degradation of a hardware component, a crystal oscillator in the control circuit of an autonomous braking system, which is in itself a CPS, is studied in detail. The impact of the degradation of the oscillator due to prolonged operation presents itself as an increase in the braking distance which is a crucial parameter for vehicles. The mechanisms of the impact are investigated in detail and a non-linear increase in the braking distance with its degradation duration of aging, i.e., temperature cycling, is also clarified. Such non-linearity information is important so that one cannot set a limit on the operation life of the CPS based on the correlation between the degradation of the oscillator and the braking distance before the onset of non-linear degradation. With a detailed understanding of the physics of failure/degradation of the oscillator, design for reliability for CPS with respect to this component can be possible. We found that the overshoot and undershoot of the PWM signal that controls the motor are the most important parameters to be guarded against.

To determine the exact time for the onset of non-linearity in the increase in the braking distance under normal operating conditions, the knowledge of the acceleration factor of the temperature cycling test performed in this work and with respect to the normal operation condition is required. Such acceleration factor can be derived either through monitoring the degradation of the equivalent electrical parameters of the crystal oscillator and thus establishing an empirical model, or it can also be derived from the physical mechanisms of the degradation of the parameters of the oscillator through detail failure analysis of the crystal degradation, and this will constitute our future work.

The advantage of this study lies in development of DfR for CPS. While this work establishes a strong foundation, it also opens up future opportunities to explore the failure physics of components in conjunction with CPS system knowledge. With the wide range of hardware components involved, future research can focus on a more detailed analysis of failure physics for components identified as critical to reliability. These critical components can be identified using advanced techniques such as Fault Tree analysis, incorporating the Birnbaum index^70^or Fussel-Vesely importance^71^ importance measures. These are promising areas for future exploration.

## Data Availability

Data will be made available on request. The corresponding author Professor Cher Ming Tan should be contacted for the Data availability.
